# Digital Mental Health Interventions in Obesity Management: A Systematic Review and Evidence-Based Recommendations

**DOI:** 10.1007/s13679-026-00735-2

**Published:** 2026-07-16

**Authors:** Gloria Marchesi, Giada Rapelli, Michelle Semonella, Alessia Bruzzese, Martina Molinaro, Gerhard Andersson, Gianluca Castelnuovo, Giada Pietrabissa

**Affiliations:** 1 Department of Psychology, Catholic University of Milan, Milan, Italy; 2 Department of Psychology, Bar-Ilan University, Ramat Gan, Israel; 3 Department of Behavioural Science and Learning, Linköping University, Linköping, Sweden; 4 Department of Clinical Neuroscience, Karolinska Institute, Stockholm, Sweden; 5 Clinical Psychology Research Laboratory, IRCCS Istituto Auxologico Italiano, Milan, Italy

**Keywords:** Digital mental health interventions, Obesity, Weight loss, Psychological outcomes, Adherence, Clinical psychology

## Abstract

**Background:**

Obesity is a complex condition encompassing behavioral, psychological, and physiological factors, frequently associated with elevated mental health burden. Digital Mental Health Interventions (DMHIs) have emerged as promising tools to enhance accessibility, personalization, and scalability of psychological care in obesity management. However, evidence on their effectiveness across delivery modalities and outcomes remains fragmented.

**Objective:**

This systematic review aimed to evaluate the effectiveness of DMHIs on obesity-related clinical, behavioral, and psychological outcomes; examine whether specific delivery modalities are differentially associated with particular outcomes; and identify methodological gaps to guide future research and implementation.

**Methods:**

A systematic search was conducted in PubMed, Scopus, PsycINFO, Cochrane Library, Web of Science, and Google Scholar. The review followed PRISMA guidelines, applying rigorous inclusion criteria and independent screening by two reviewers. Quality appraisal was performed using the Cochrane Risk of Bias Tool (RoB 2.0), and studies rated as high risk of bias were excluded. Due to heterogeneity in study design and outcomes, data were synthesized narratively, and no claims of statistical superiority between modalities were made.

**Results:**

Thirty-eight randomized controlled trials were included. DMHIs effectively targeted behavioral and psychological aspects of obesity when based on evidence-based psychotherapeutic frameworks - often independently of weight-related improvements. No single delivery format emerged as universally superior; rather, each modality appeared to serve distinct therapeutic purposes. Outcomes were more favorable in interventions that incorporated human guidance, although this finding should be interpreted cautiously because of heterogeneity and the absence of meta-analytic comparisons.

**Conclusions:**

Digital mental health approaches—particularly when integrated into stepped-care or hybrid models—represent scalable, person-centered strategies to improve both physical and emotional well-being in adults with obesity.

**Supplementary Information:**

The online version contains supplementary material available at 10.1007/s13679-026-00735-2.

## Introduction

Obesity is a chronic, relapsing condition, typically defined as a body mass index (BMI) ≥ 30 kg/m², and characterized by the abnormal or excessive accumulation of adipose tissue. It substantially increases the risk of numerous severe comorbidities, including type 2 diabetes, cardiovascular and cerebrovascular diseases, hypertension, dyslipidemia, certain cancers, musculoskeletal disorders, and arthritis [1, 2].

The global prevalence of obesity has risen at an alarming pace. In 2022, more than 890 million adults - approximately 16% of the world’s population—were living with obesity [3]. Projections from the GBD 2025 Obesity Collaborators [4] estimate that by 2050, this number will escalate to 3.8 billion adults, representing nearly 60% of the global adult population. Such trends pose an unprecedented challenge for healthcare systems, the majority of which are inadequately resourced to address the complex medical and psychosocial needs associated with obesity. According to the World Obesity Atlas [5], only 7% of countries report health systems prepared to manage the obesity epidemic, while almost two-thirds lack comprehensive national strategies to mitigate its impact.

Obesity is the result of multifactorial and dynamic interactions among biological, environmental, socioeconomic, and behavioural determinants [6]. Importantly, psychological factors - including depression, anxiety, disordered eating, perceived stigma, discrimination, and chronic stress - play a pivotal role in both the onset and maintenance of obesity [7]. Conversely, obesity is strongly associated with poor mental health outcomes, including an increased risk of depressive symptoms and mood disorders [8]. These bidirectional associations highlight the need for comprehensive and integrated treatment approaches. Interventions that target psychological as well as physical health outcomes are essential to promote sustainable weight management and improve quality of life (QoL) [9].

Clinical psychological interventions are a cornerstone of multidisciplinary obesity treatment. They provide individuals with strategies to manage psychological distress, modify maladaptive cognitions, develop self-regulation skills, and replace unhealthy behaviours with more adaptive patterns [10, 11]. Traditional face-to-face interventions, including Cognitive Behavioural Therapy (CBT), Acceptance and Commitment Therapy (ACT), and Mindfulness-Based Interventions (MBIs), have shown robust efficacy in promoting behavioural change and supporting weight loss [12, 13].

Despite short-term efficacy, conventional interventions often fail to deliver durable benefits. Limited access to structured programs, the high financial and organizational burden of in-person care, and declining adherence over time are major barriers. Moreover, weight regain after initial loss is common, reflecting the complex, multifactorial, and relapsing nature of obesity and the difficulty of sustaining behavior change. These limitations underscore the need for innovative, scalable, and sustainable approaches that complement and enhance standard treatments [14].

In this context, Digital Mental Health Interventions (DMHIs) have emerged as promising strategies to address these gaps. Delivered through mobile applications, web-based platforms, telemedicine, virtual reality (VR), and artificial intelligence (AI)-driven systems, these tools provide potentially scalable, and accessible options capable of reaching larger and more diverse populations; however, their cost-effectiveness requires further empirical confirmation in obesity populations [15, 16]. Importantly, DMHIs often integrate evidence-based psychological and behavioral frameworks - including CBT, MBIs, psychoeducation, and ACT - into digital formats. They also employ a variety of behavior change techniques, such as self-monitoring, goal setting, action planning, and personalized feedback, to actively engage users in the change process [17–19]. Structured exercises, ranging from digital food diaries and guided breathing sessions to interactive reflective worksheets, are frequently incorporated to foster self-awareness, enhance emotional regulation, and strengthen adherence to lifestyle changes. By providing continuous, flexible, and personalized support, DMHIs represent a potentially transformative avenue in overcoming the persistent challenge of obesity.

However, heterogeneity in study designs, delivery modalities, and outcome measures hampers drawing robust conclusions about their effectiveness and scalability.

An integrated synthesis across these outcomes is therefore essential to capture the full clinical potential of DMHIs and to clarify how they contribute to overall well-being of people with obesity. Such evidence will help tailor interventions to patient needs, optimize resource allocation, and inform the design of hybrid or stepped-care models that combine human and digital components effectively.

Accordingly, this systematic review aims (1) to synthesize evidence on the effectiveness of DMHIs for obesity-related outcomes spanning clinical psychological (i.e., depression, anxiety, stress, QoL, disordered eating), and behavioral endpoints; (2) to narratively examine patterns of association between delivery modalities (e.g., apps, web-based platforms, telephone, videoconferencing, VR/IVR) and specific outcomes, without inferring statistical superiority across modalities; and (3) to identify gaps to guide future research and clinical implementation.

## Materials and Methods

This review was conducted in accordance with the Preferred Reporting Items for Systematic Reviews and Meta-Analyses (PRISMA) guidelines [20]. The completed PRISMA Checklist for the reporting of this systematic review is provided in Online Resource 1. The protocol for this study was registered with the International Prospective Register of Systematic Reviews (PROSPERO), nr. CRD420251010692. Trial Registration: PROSPERO ID CRD420251010692.

### Literature Search

A comprehensive literature search was conducted across six databases – Scopus, PubMed, Google Scholar, PsycINFO, Cochrane Library, and Web of Science – from November 3 to November 13, 2025. Under the PICO framework (Patient problem or population; Intervention; Comparison or control and Outcome) [21], the search strategies included the following terms: (obesity) OR (obes*) OR (BMI) AND (Web-based intervention) OR (Internet-based intervention) OR (Online intervention) OR (Digital intervention) OR (eHealth) OR (mHealth) OR (Telemedicine) OR (Telehealth) OR (Mobile application) OR (Digital health) OR (Internet-based therapy) OR (Mobile health) OR (Online therapy) OR (Computerized therapy) AND (Psychotherapy) OR (Therapy) OR (Cognitive behavioural therapy) OR (CBT) OR (Behaviour therapy) OR (Mindfulness) OR (acceptance and commitment therapy) OR (ACT) OR (Psychological intervention) OR (Psychological support) OR (Self-management program) OR (Psychoeducation) AND (Guided intervention) OR (Guided self-help) AND (waiting list) OR (standard treatment) OR (treatment-as-usual) OR (TAU) OR (other intervention) AND (psychological well-being) OR (mental health) OR (emotional well-being) OR (psychological distress) OR (quality of life) OR (depression) OR (anxiety) OR (stress) OR (self-esteem) AND (randomized controlled trial) OR (RCT) OR (clinical trial) OR (random allocation). The search terms were strategically combined using Boolean operators and truncation symbols, with the search syntax adapted as needed for each database.

### Inclusion and Exclusion Criteria

Eligibility was limited to articles that satisfied the following criteria: (1) randomized controlled trials (RCTs), (2) participants aged over 18 years, (3) sample’s mean BMI ≥ 30 kg/m², (4) studies assessing the efficacy of digital mental health interventions for obesity management, (5) included at least one psychological outcome, and (6) trials rated as having either a low risk of bias or some concerns according to the Cochrane Risk of Bias Tool (RoB 2.0) [22]. Studies were excluded if rated as high risk of bias [22] or did not involve human participants. No restrictions were applied regarding language or year of publication.

### Selection Process

Two researchers (authors A.B. and M.M.) independently assessed the eligibility of the articles by first reviewing titles and abstracts, followed by a full-text evaluation. Records were managed in a shared screening file and screened independently by the two reviewers at title/abstract and full-text stages; disagreements were documented and resolved by consensus or, when necessary, in consultation with a third team member (author G.P.). Additionally, the reference lists of the retrieved systematic reviews and included studies were manually screened to identify further relevant publications. The review team included at least one person (authors G.P. and G.R.) with methodological expertise in conducting systematic reviews, as well as at least two experts on the topic under review (authors G.C., G.P., and M.S.) [23].

**Fig. 1:**
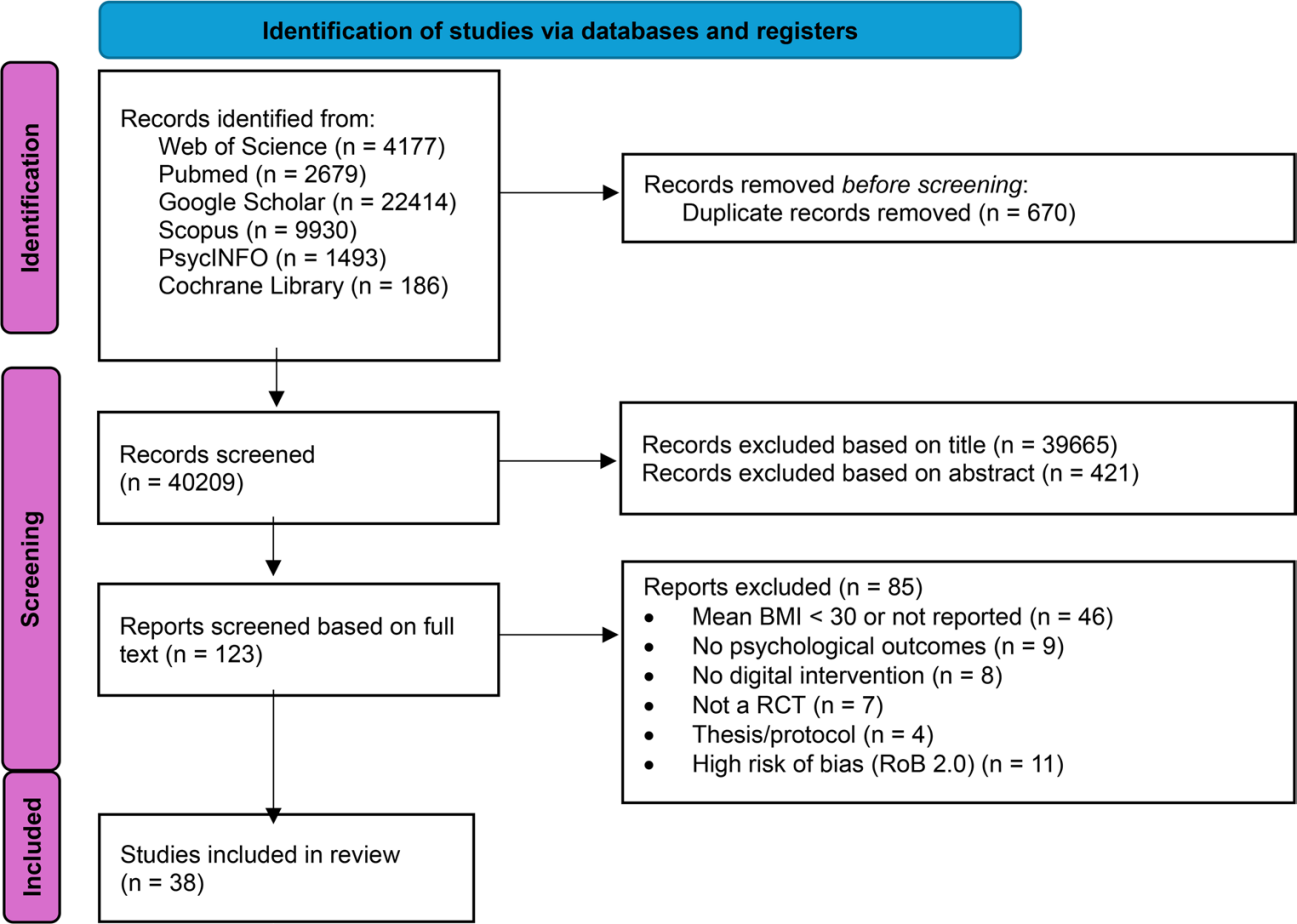
The PRISMA flowchart

In accordance with the PRISMA guidelines [24], Fig. 1 provides the detailed process of study selection through a structured flowchart.

### Data Extraction

Reviewers A.B. and M.M. independently extracted the following data: author and year of publication, country, BMI, sample size, participants’ age and gender, type and duration of the intervention, and main outcomes. Any disagreements were resolved by consensus and consultation with a third researcher (Author G.P.).

### Appraisal of Methodological Quality and Risk of Bias

The methodological rigor of the included RCTs was evaluated using the Cochrane Risk of Bias Tool (RoB 2.0) [22]. This instrument examines five critical domains: (1) randomization process, (2) deviations from the intended intervention, (3) missing outcome data, (4) measurement of the outcome, and (5) selection of the reported results. Each domain is assessed through signaling questions designed to support a structured appraisal of the available evidence. Responses are provided on a five-point scale (“Yes,” “Probably Yes,” “Probably No,” “No,” or “No Information”), depending on certainty and data availability. These responses feed into an algorithm that classifies each domain as having a low risk of bias, some concerns, or a high risk of bias. An overall judgment is then assigned to each trial: “low risk” if all domains are rated low, “some concerns” if at least one domain raises issues without reaching high risk, and “high risk” if at least one domain is rated high or if multiple domains collectively undermine confidence in internal validity. Methodological quality assessments were independently conducted by two reviewers (A.B. and M.M.), with disagreements resolved by a third researcher (G.P.). The results are summarized in the Online Resource 2.

### Data Analysis and Synthesis

Initially, RCTs were examined, and pertinent details were extracted and documented. The extracted data were used to produce a narrative summary of the effects of digital psychological interventions for obesity management.

## Results

### Study Selection

The systematic search retrieved 40,879 records. After removal of duplicates (*n* = 670), 39,665 records were excluded based on title screening. Abstract evaluation of the remaining 544 articles resulted in the exclusion of 421 studies. A total of 123 full-text articles were assessed for eligibility. Among these, 46 studies were excluded because the mean BMI of participants was either not reported or below 30 [25–70], 9 records were omitted for not including psychological outcomes [71–79], and 8 articles were excluded as they did not focus on digital interventions [80–87]. Further, seven records were omitted because they were not RCTs [88–94], and 4 articles were excluded because they were theses or articles that had not undergone peer review [95–98]. The remaining 49 studies underwent methodological quality assessment using the RoB 2.0 tool, which led to the exclusion of 11 studies [99–109] rated at high risk of bias. Thus, 38 studies met all inclusion criteria and were included in the present systematic review [110–147].

### Study Characteristics

The included studies were published between 2001 [137] and 2025 [113] and were conducted in the United States (*n* = 15) [111, 112, 121, 122, 124–126, 128, 129, 132, 135, 143, 145–147], Australia (*n* = 7) [114, 117–119, 130, 136, 144], Canada (*n* = 4) [115, 134, 139, 140], the United Kingdom (*n* = 3) [127, 131, 138], Germany (*n* = 2) [141, 142], Italy (*n* = 3) [113, 116, 137], Finland (*n* = 1) [123], Greece (*n* = 1) [120], Iran (*n* = 1) [110], and Turkey (*n* = 1) [133].

Notably, two records [117, 118] were based on the same participant sample but were both included because they reported distinct, non-overlapping outcomes relevant to the review objectives. To avoid double-counting, demographic and baseline characteristics were extracted only once and not duplicated in the descriptive summaries.

In total, the included studies comprised 5,553 participants, with sample sizes ranging from 18 [145] to 667 [125]. Across studies, the mean age of participants was 42.5 years, with ages ranging from 18 to 86 years [146]. The mean BMI was 34.4 kg/m², with reported values spanning from approximately 30.0 kg/m² [110] to 53.1 kg/m² [115]. Study details and participant characteristics are presented in Online Resource 3.

### Delivery Methods of Digital Mental Health Interventions in Obesity

Across the included studies, web-based platforms or webpages/websites were the most commonly used delivery modality, appearing in 16 studies [112, 117, 118, 120, 125–128, 130–132, 141, 143–146]. Eleven interventions were delivered via mobile applications [110, 111, 113, 114, 121–124], with some interventions integrating apps with additional digital tools such as websites [129, 135] or telephone support [119]. Telephone-based interventions were also common, being adopted in six trials [115, 134, 136, 138–140], while an interactive voice response (IVR) system was tested in a single study by Zoellner et al. (2018) [147]. Moreover, video conferencing [133, 142] and VR platforms were used in two studies [116, 137]. Details are reported in Online Resource 4.

#### Web-Based Platforms and Website Interventions

Among the 17 studies that delivered interventions via web-based platforms or websites for individuals with obesity, 7 records were judged to have an overall low risk of methodological bias [117, 118, 125, 130, 131, 141, 144], while nine raised some concerns [112, 120, 126–128, 132, 143, 145, 146], primarily related to the randomization process [112, 126, 128, 132, 143, 145, 146], deviations from the intended interventions [132, 145], missing outcome data [127], or selection of reported results [120]. All studies were rated as having a low risk of bias in the measurement of the outcomes domain.

Across studies, experimental groups typically received web-based or online interventions, compared with control conditions that included treatment-as-usual (TAU) [112, 120, 125, 127], enhanced usual care (EUC) [146], waitlist (WL) [117, 118, 126, 130, 131, 141, 144], and alternative digital, face-to-face (F2F) or self-guided programs [128, 132, 143, 145].

The experimental interventions incorporated diverse components: several studies emphasized cognitive strategies, such as cognitive restructuring [117, 118, 144, 145], ACT skills [126], reflective exercises or activity diaries [125, 131, 141], and mindfulness [117, 118, 144, 145]; others primarily targeted behavioral activation [117, 118, 120, 127, 128, 131] and relapse prevention [144, 145] or focused on motivational strategies [112, 145]. Further records incorporated psychoeducational content related to nutrition and healthy lifestyle behaviors [112, 117, 118, 120, 126–128, 130–132, 141, 145]. Self-monitoring and goal-setting elements were also implemented [120, 128, 130, 132, 146]. Additional features were designed to enhance engagement and interactivity, including online support [143], group chats [128], individualized feedback [141], social forums, gamification, and video coaching [125]. In terms of delivery format, several programs were self-guided [117, 118, 120, 125, 127, 130, 132, 143, 144], whereas others included professional guidance, provided by therapists [126, 141], medical assistants [112], health coaches [131, 146], or combinations of dietitians and therapists [128], or mental health counselors and dietitians [145]. The theoretical approaches underpinning the interventions were heterogeneous: most studies were grounded on CBT principles [128, 132, 141, 143, 145, 146], sometimes combined with elements of Social Cognitive Theory (SCT) [144]; others relied on SCT only [117, 118, 127, 130], ACT [126, 131], or Motivational Interviewing (MI) techniques [112]. Additional theoretical foundations included the Self-Regulation Approach (SRA) [120] and the Self-Enhancement Theory (SET) [125]. Program duration varied across studies: short-term interventions lasted 30 days to 3 months [112, 117, 118, 125, 126, 130–132, 144, 145]; medium-term programs extended 3–6 months [120, 141, 146]; longer-term interventions lasted up to 12 months [127, 128, 143]. Frequency and intensity of sessions or activities also varied: some protocols emphasized daily tasks or self-monitoring [120, 141, 143], whereas most records employed weekly scheduled activities [112, 126–128, 131, 144–146, 148]. Follow-up assessments were also heterogeneous: some studies included only baseline and post-treatment evaluations [132, 145], whereas others incorporated mid-treatment checkpoints [143] or combined mid-treatment assessments with longer-term follow-ups [127, 128]. Overall, follow-ups ranged from 2 months [112, 125, 126] and 6 months [117, 118, 120, 130, 144] up to 12 months [131, 141, 146].

#### App-Based Interventions

Five [111, 114, 119, 124, 135] out of the 11 studies evaluating app-based interventions for obesity management were judged to have an overall low risk of methodological bias. The remaining 6 studies raised some concerns [110, 113, 121–123, 129], primarily related to the measurement of outcomes domain [113], the randomization process [123], deviations from the intended interventions [121, 129], and selective reporting of results [110, 122]; in addition Hildebrandt et al. (2020) also presented concerns regarding missing outcome data [122]. Bertoli et al. (2025) [113] was the only study rated as having some concerns across all risk-of-bias domains.

Across studies, app-based psychological or behavioural interventions were compared with different controls, including TAU [110, 111, 113, 122, 124], WL [110, 119, 123, 135], or alternative digital/behavioral programs [114, 121, 129].

Components of the experimental included cognitive-behavioral strategies - combining psychoeducational modules with self-monitoring tools, assignments, and feedback [110, 122]; behavioral tracking and social support features, such as diet and physical activity monitoring, weight loss tracking, and peer interaction [119, 121, 124, 129]; or motivational strategies [111, 114]. Additional studies integrated acceptance- and mindfulness-based techniques, including value clarification, digital meditation, and healthy eating guidance [113, 123, 135]. In terms of delivery format, four interventions were self-guided [113, 114, 119, 121, 124], while others were supported by professional guidance, including psychologists [111, 123, 129], doctoral students in clinical psychology [110], or health coaches [122]. A hybrid delivery format was also reported, in which participants alternated between self-directed app use and scheduled check-ins with healthcare professionals [135]. The theoretical underpinnings of the interventions varied across studies and included CBT [110, 111, 119, 122], SCT–based models [121, 129], behavioral approaches [124], ACT [123], or mindfulness-based frameworks [113, 135]. In addition, one study was grounded in the Health Action Process Approach (HAPA) [114].

The duration of the app-based interventions ranged from 6 to 8 weeks [110, 114, 123, 135] to 12 weeks [121, 122], 16 weeks [129], 24 weeks [113, 119, 124] or longer [111]. In terms of frequency of use, some studies encouraged daily engagement with the app [110, 124]. Others structured the intervention in weekly sessions [129], or combined different frequencies such as daily and weekly [121, 135], weekly and biweekly [111], or daily, weekly, and biweekly sessions [113, 122].

Follow-up assessments were heterogeneous. Some studies included only baseline and post-treatment evaluations [110, 113, 121, 123, 135], whereas others incorporated mid-treatment checkpoints [114] or longer-term assessments, including 6-month [124], 9-month [123], or 12-month [111, 119, 122, 129] follow-ups.

#### Telephone-Based Interventions or Interactive Voice Response (IVR) Systems

Of the studies delivering telephone-based [115, 134, 136, 138–140] or IVR system [147] interventions, 4 records presented an overall low risk of methodological bias [115, 136, 138, 140], while the remaining 3 showed some concerns in the randomization process [134, 139, 147] in the randomization process; additionally, Pearson et al. (2012) [134] raised concerns regarding deviations from the intended interventions.

In these RCTs, experimental groups received structured behavioral or psychological interventions delivered via telephone or IVR, compared with TAU controls [115, 136, 138–140], or alternative digital/behavioral programs [134, 147].

The experimental interventions incorporated diverse components, including cognitive restructuring, problem-solving, and behavioral activation [115, 139, 140, 147]. Some interventions emphasized self-monitoring and feedback, such as tracking goal progress and providing reinforcement [138, 139]. One study included coaching [134], while another specificcaly targeted emotional regulation and eating behaviors [140]. Professionals who guided the interventions were therapists [116, 138–140], dietitians [136], or life coaches [134], though certain components were fully or partially self-directed [134, 147]. The theoretical underpinnings encompassed CBT [115, 134, 139, 140, 147], self-regulation theory [138], and SCT [136]. Program duration varied markedly: short-term interventions lasted 6 weeks [115], 10 weeks [139, 140], or 12 weeks [134]; medium-term programs ranged from 6 months [136] to 12 months [138], and the longest-term intervention spanned 18 months, including active and maintenance phases [147]. Follow-up schedules were heterogeneous, with some studies assessing only baseline and post-treatment [115, 136, 138], one including a mid-treatment checkpoint [134], and others evaluating outcomes at 3 months [140], 6 months [139], and 18 months [147] post-treatment.

#### Video Conferencing Platforms

Both studies, which used video conferencing platforms to deliver DMHIs for obesity [133, 142], presented some concerns regarding the selection of reported results. In addition, Ozturk and Duruturk (2022) [133] presented concerns related to the randomization process and deviations from the intended interventions.

In both trials, the experimental intervention was therapist-guided and compared with a WL control [133] or standard post-surgical visits [142].

Ozturk and Duruturk (2022) [133] implemented a clinical telerehabilitation model including live remote warm-up exercises, trunk stabilization exercises, and breathing exercises. The intervention comprised three 45-minute sessions per week for 6 weeks, and participants were evaluated at baseline and post-treatment. Instead, Wild et al. (2015) [142] tested a 1-year videoconferencing-based psychoeducational program comprising five preoperative face-to-face sessions, six videoconferencing sessions, and three postoperative in-person group meetings addressing nutrition and eating behavior, stress and emotional eating, encouragement of physical activity, coping with social conflict and stigma, body image and self-esteem, self-care, and mindfulness training. Participants were assessed at 1-, 3-, 6-, and 12-month post-intervention.

#### Virtual Reality

Of the two studies investigating VR-based interventions for obesity [116, 137], one RCT [116] was rated as having a low overall risk of bias, whereas the other [137] raised some concerns related to the randomization process and deviations from the intended interventions.

In both trials, participants in the experimental groups received 6-week therapist-guided VR interventions, incorporating a range of cognitive-behavioral and experiential components. Particularly, Cesa et al. (2013) [116] compared two experimental conditions: a VR-enhanced CBT (ECT), including body image rescripting, VR exposure scenes, emotional and cognitive work, role-play, and problem-solving, and a CBT program focused on self-monitoring, behavioral coping strategies, and relapse prevention with a TAU control [116]. Riva et al. (2001) [137], instead, implemented the so-called VREDIM protocol, consisting of seven sessions of virtual reality exposure, integrated with cognitive-behavioral techniques, body image rescripting, motivational interviewing, and assertiveness training, alongside low-calorie diet guidance and physical training. In both studies, participants were evaluated at baseline and post-treatment.

### Impact of Digital Tools for Mental Health Interventions on Clinical, Behavioural, and Psychological Outcomes

#### Web-Based Platform and Website Interventions

Evidence from web-based interventions showed mixed effects on weight-related outcomes, although several studies reported significant reductions compared with controls. For example, Naparstek et al. (2017) [132] observed greater post-treatment weight loss in the web intervention group; Young et al. (2021) [144] and Drew, Morgan, and Young (2022) [118] reported greater weight reduction in the intervention group over 3–6 months. Barnes et al. (2014) [112] found that the experimental condition achieved greater weight loss than TAU from baseline to 12 months and from 12 to 24 months. In contrast, the Motivational Interviewing Condition (MIC) did not differ from either TAU or NPC. Likewise, Morgan et al. (2013) [130] documented significant weight reductions at 3 and 6 months in both web-based conditions versus WL, with no differences between the two active formats.

By contrast, some RCTs reported no difference in weight change between intervention and control groups in body weight or BMI at post-treatment, 6 months, or 12 months [120, 128, 131, 141, 146]. Similarly, Yu et al. (2021) [145] observed no between- or within-group differences over 12 months. In addition, Womble et al. (2004) [143] reported a detrimental effect, with the intervention group achieving less weight loss than the control at both short- and long-term follow-ups.

Findings on changes in maladaptive eating were also inconsistent across studies. Wagner et al. (2016) [141] reported clear reductions of objective binge-eating episodes (OBEs) and improvements in the Eating Disorder Examination Questionnaire (EDE-Q) subscales (Restraint, Weight Concern, Shape Concern, Eating Concern), Mueller et al. (2022) [131] observed healthier eating profiles - higher Cognitive Restraint and lower Uncontrolled Eating on the Three-Factor Eating Questionnaire (TFEQ), while Levin et al. (2021) [126] showed improvements in the TFEQ and higher Healthy Eating Index (HEI) scores among participants receiving the intervention, although these effects were not maintained at the 16-week follow-up. Extending this evidence, Drew, Morgan, Collins et al. (2022) [117] and Drew, Morgan, and Young (2022) [118] documented reduced intake of energy-dense, nutrient-poor (EDNP) foods and lower risky alcohol use on the Alcohol Use Disorders Identification Test–Consumption (AUDIT-C), and Morgan et al. (2013) [130] reported better portion control and dietary behaviors. All effects were observed in the experimental groups compared with WL controls.

Evidence was more nuanced for eating-disorder psychopathology in Yu et al. (2021) [145]. However, body-shape concerns and global EDE-Q psychopathology improved, showing some advantages for the videoconference-based format, whereas uncontrolled and emotional eating improved in the face-to-face arm.

Moreover, Womble et al. (2004) [143] reported within-group increases in Cognitive Restraint and decreases in Disinhibition and Hunger on the Eating Inventory (EI) for the experimental group, but found no significant between-group differences. Last, Micco et al. (2007) [128] observed lower daily caloric intake across groups (indicating dietary improvement rather than a targeted reduction in maladaptive eating), while McConnon et al. (2007) [127] detected no significant changes in dietary habits from baseline to 12-month post-treatment.

Concerning mental health outcomes, several studies reported beneficial effects of the interventions on depressive symptomatology. Naparstek et al. (2017) [132] and Young et al. (2021) [144] observed significant reductions in depressive symptoms in the intervention group at 3-month post-treatment, as measured by the Center for Epidemiologic Studies Depression Scale (CES-D) and the Patient Health Questionnaire (PHQ-9), respectively. In Young et al. (2021) [144], a higher proportion of participants in the intervention group achieved a clinically meaningful improvement (≥ 50% reduction in depressive symptoms), with effects maintained at 6-month follow-up and additional gains observed on the Beck Depression Inventory (BDI) and the Male Depression Risk Scale–22 (MDRS-22). In contrast, effects on anxiety were generally smaller, as assessed by the Generalized Anxiety Disorder–7 (GAD-7). Similarly, Barnes et al. (2014) [112] and Wagner et al. (2016) [141] documented lower depressive symptomatology at post-treatment in the intervention group compared with the TAU and WL controls, respectively, using the BDI; with Wagner et al. (2016) [141] also documenting effects on anxiety on the Symptom Checklist-90-Revised (SCL-90-R). Similarly, Womble et al. (2004) [143] found reductions in depressive symptoms at 52 weeks, accompanied by partial improvements in gains in health-related quality of life (HRQoL) assessed with the Short Form Health Survey–36 (SF-36). Conversely, Yu et al. (2021) [145] and Zamorano et al. (2021) [146] reported no significant between-group differences for depressive symptoms (assessed with the Beck Depression Inventory-II, BDI-II, and PHQ-9, respectively) or HRQoL measured by the Impact of Weight on Quality of Life-Lite, IWQOL-Lite, and the SF-12. Similarly, Mueller et al. (2022) [131] found no significant effects on depression (Patient Health Questionnaire-8, PHQ-8), anxiety (GAD-7), or perceived stress (Perceived Stress Scale-4, PSS-4), although improvements were observed in psychological flexibility, as measured by the Acceptance and Action Questionnaire for Weight–Revised (AAQW-R), and in capability and well-being, assessed using the ICEpop CAPability measure for Adults (ICECAP-A). Regarding well-being–related outcomes, Lee et al. (2021) [125] reported selective improvements, with significant increases in community and occupational well-being actions, while no effects were observed in the physical, interpersonal, or economic domains. The same study also documented enhanced well-being–related self-efficacy, as measured by the Well-Being Actions Self-Efficacy (WBASE) scale. With respect to HRQoL, improvements were reported by Morgan et al. (2013) using the SF-12 [130]; however, McConnon et al. (2007) [127] did not detect significant between-group differences when HRQoL was assessed using the EuroQol-5 Dimensions (EQ-5D).

Likewise, Goulis et al. (2004) [120] detected no significant effects on HRQoL (SF-36 and EQ-5D) but high satisfaction on the Telemedicine Satisfaction Questionnaire (TSQ). In addition, Levin et al. (2021) [126] observed improvements in general mental health, assessed with the General Health Questionnaire (GHQ), alongside reductions in weight self-stigma measured by the Weight Self-Stigma Questionnaire (WSSQ). Beyond emotional and symptom-based outcomes, Drew, Morgan, Collins et al. (2022) [117] and Drew, Morgan, and Young (2022) [118] reported greater gains in cognitive flexibility (Cognitive Flexibility Inventor, CFI) and perceived physical strength (Physical Self Perception Profile-Short Form, PSPP-SF) in the experimental condition compared with WL controls; and Micco et al. (2007) [128] documented higher perceived social support in the internet-only group at both 6-month and 12-month follow-ups, whereas no between-group differences were observed in perceived therapist support.

Overall, dropout varied considerably across studies, ranging from 3.4% [112] to 55.6% [145].

The results concerning the effects of DMHIs on primary and secondary outcomes are reported in Online Resource 5.

#### App-Based Interventions

Weight-related outcomes were heterogeneous across studies. Significant between-group advantages for the intervention were reported in some trials, with greater BMI or weight reductions compared with control conditions [110, 111, 121]. Bertoli et al. (2025) [113] and Monroe et al. (2019) [129] found clinically meaningful weight loss in both intervention and comparison groups, with no significant between-group differences at post-treatment or follow-up; notably, Bertoli et al. (2025) [113] also reported higher adherence in the intervention group, alongside significant reductions in BMI and waist circumference at 6 months. Other trials reported no significant differences in BMI [135] or weight loss [114, 119], and Laing et al. (2014) [124] reported greater weight loss in the control group than in the experimental condition at 6-month follow-up. Only one study [123] assessed inflammatory and stress biomarkers: interleukin 1 receptor antagonist (IL-1Ra) decreased significantly overall; high-sensitivity C-reactive protein (hsCRP) reductions were confined to the face-to-face condition; cortisol and dehydroepiandrosterone sulfate (DHEAS) did not change significantly.

Regarding maladaptive eating behaviors, Hildebrandt et al. (2020) [122] reported a significant reduction in objective binge eating days, assessed through structured clinical interviews and the EDE-Q, which was maintained at 9 months post-intervention. Similarly, Radin et al. (2023) [135] evaluated pre-post changes in binge eating frequency using the Questionnaire on Eating and Weight Patterns-5 (QEWP-5), in combination with adherence metrics, and found improvements in eating-related behaviors and treatment engagement. Moreover, concerning mental health dimensions, Radin et al. (2023) [135] reported significant reductions in perceived stress (PSS) favoring the intervention, whereas no effects were observed for food craving tolerance, measured with the Food Craving Questionnaire (FCQ), at 2 months. In contrast, both Abedishargh et al. (2021) [110] and Duncan et al. (2020) [119] found no significant between-group differences in depression, anxiety, and stress, measured with the Depression Anxiety Stress Scales (DASS), and Altazan et al. (2019) [111] similarly reported null between-group effects for depressive symptoms (BDI-II) and HRQoL (SF-12). Conversely, Hildebrandt et al. (2020) [122] observed reductions in depressive symptoms (PHQ-8) and improvements in general QoL following a 3-month intervention, although these effects were not sustained at 9-month follow-up. Last, Jarvala-Reijonen et al. (2020) [123] documented significant improvements in both general and weight-related psychological flexibility (Acceptance and Action Questionnaire-II, AAQ-II and AAQW) over time among participants receiving app-based ACT. Overall, dropout varied considerably across studies, ranging from 10% [135] to 38.2% [122]. Interventions involving therapist or health coach support, such as those of Altazan et al. (2019) [111] and Abedishargh et al. (2021) [110], generally reported lower dropout compared with fully self-guided programs.

#### Telephone-Based Interventions or Interactive Voice Response (IVR) Systems

Telephone-based interventions generally yielded positive within-group changes in weight-related outcomes, although between-group differences were less consistent. Reeves et al. (2017) [136] observed significant weight loss, waist circumference, and fat mass reductions compared with TAU. Pearson et al. (2012) [134] reported significant within-group weight reductions over time but no between-group differences. Similarly, Sniehotta et al. (2019) [138] found improvements in healthy behaviors within the intervention group without significant group differences at 12 months, while Sockalingam et al. (2023) [140] noted modest mean weight reductions in the intervention group. Only one study [147] examined reductions in sugar-sweetened beverage (SSB) intake, reporting decreases in the intervention groups compared with controls; however, these changes did not lead to significant improvements in weight or BMI.

Three studies reported significant reductions in binge eating and emotional eating, assessed with the Binge Eating Scale (BES) and Emotional Eating Scale (EES), respectively [115, 139, 140]. Participants in the tele-CBT groups showed improvements over time, whereas control participants remained stable or worsened. The same pattern was observed for depressive (PHQ-9) and anxiety (GAD-7) symptoms. Regarding health-related quality of life (HRQoL), Cassin et al. (2016), Cassin et al. (2016) [115], Pearson et al. (2012) [134], and Reeves et al. (2017) [136] reported no significant improvements using the SF-36, and Zoellner et al. (2018) [147] found null results with the Centers for Disease Control Health-Related Quality of Life (CDC HRQoL-14). In contrast, Sniehotta et al. (2019) [138] documented improvements in HRQoL assessed with the EQ-5D. In addition, within-group improvements favoring the intervention were observed for self-esteem (Rosenberg Self-Esteem Scale, RSES) [134], body image (Body Image and Relationships Scale, BIRS) [136], and perceived social support (Enhancing Recovery in Coronary Heart Disease Social Support Inventory, ENRICHD-SSI) [138], though no significant between-group differences were found across studies. Dropout rates ranged from 6% [139] to 42.3% [134].

#### Video Conferencing Platforms

The two studies examining the effectiveness of videoconference-based psychological or rehabilitative interventions on physical and psychological outcomes yielded inconsistent findings [133, 142]. In Ozturk and Duruturk (2022) [133], participants in the intervention arm showed significant advantages over WL controls in health-related physical fitness, measured with the Senior Fitness Test Protocol (SFTP), and in HRQoL (SF-36) 6 weeks after treatment initiation. In Wild et al. (2015) [142], no significant between-group differences emerged for HRQoL or self-efficacy (General Self-Efficacy Scale, GSE) at either 6-month or 1-year follow-ups. Also, although both groups experienced substantial weight reduction, the decrease did not differ significantly between them. Notably, among participants with clinically significant depressive symptoms at baseline (PHQ-9 ≥ 10), the intervention group demonstrated greater improvements in physical and mental functioning, lower depressive symptomatology, and reduced eating-related pathology as assessed by the Eating Disorder Examination (EDE) at follow-up, relative to controls.

Overall, dropout rates were minimal (0–6%) [133, 142].

#### Virtual Reality

Across both studies [116, 137], participants allocated to VR-based interventions showed greater mean weight loss compared with treatment-as-usual (TAU) controls; however, no statistically significant between-group differences were observed. In Cesa et al. (2013) [116], all study groups experienced weight loss from baseline to 6 months. During the subsequent follow-up period (6–12 months), weight regain was observed in the control group, whereas participants in the experimental conditions (CBT and Enhanced Cognitive Behavioural Therapy, ECT) maintained their weight loss, with the ECT group demonstrating an additional significant reduction. Significant within-group improvements in eating-related behaviors were observed, although no between-group differences were detected. Cesa et al. (2013) [116] reported remission of binge-eating episodes at post-treatment in all groups, followed by an increase at follow-up. Similarly, Riva et al. (2001) [137] found higher scores on the DIET Positive Social subscale in the VR group compared with controls, but a non-significant reduction in overeating (DIET Overeating subscale).

Regarding psychological outcomes, Riva et al. (2001) [137] reported a significant reduction in anxiety (State-Trait Anxiety Inventory, STAI), with greater improvement in the VR group compared with controls. Assertiveness (Assertion Inventory, AI) increased in both groups, showing larger between-group effects favoring VR, whereas no significant differences emerged for self-efficacy (Weight Efficacy Life-Style Questionnaire, WELSQ) or readiness to change (University of Rhode Island Change Assessment, URICA), despite within-group improvements in WELSQ for VR participants. Concerning body image, Cesa et al. (2013) [116] observed a significant reduction in body image avoidance (Body Image Avoidance Questionnaire, BIAQ) and non-significant changes in body satisfaction (Body Satisfaction Scale, BSS) within the ECT group. Similarly, Riva et al. (2001) [137] found no significant between-group differences in BSS scores. Attrition was 33.4% at 12-month follow-up in Cesa et al. (2013) [116], while Riva et al. (2001) [137] did not report dropout rates.

## Discussion

The present review provides an updated synthesis of evidence on the effectiveness of DMHIs for adults with obesity, examining the impact of multiple delivery modalities across clinical, behavioural, and psychological outcomes. Overall, the findings suggest that DMHIs can produce meaningful benefits when grounded in evidence-based psychotherapeutic frameworks. However, the magnitude and consistency of these effects appear to vary substantially depending on the delivery modality. In the narrative synthesis, web-based interventions showed the most consistent pattern of favorable findings across several outcomes; however, because no meta-analysis or network meta-analysis was performed, this observation should not be interpreted as evidence of statistical superiority over other modalities. These interventions demonstrated reductions in weight-related outcomes [112, 118, 130, 132, 144], improvements in maladaptive eating behaviors such as binge eating and disinhibited eating [126, 131, 141], and reductions in depressive symptoms [132, 141, 143, 144]. These results highlight the potential of structured, web-based programs to simultaneously target clinical, behavioral, and psychological domains in adults with obesity. App-based interventions also showed promising effects, particularly in reducing binge eating [122, 135], enhancing psychological flexibility [123], alleviating stress [135], and supporting engagement and adherence [113, 135]. Nevertheless, evidence for their impact on weight loss [114, 119, 124] and broader psychological outcomes was more variable, suggesting that while apps can be useful tools for specific behavioral targets, their effectiveness may depend on the presence of guidance or feedback, specific design features, and users’ levels of digital literacy. Telephone- and videoconferencing-based interventions primarily demonstrated within-group improvements, with limited evidence of superiority over control conditions. This pattern suggests that these modalities may be more appropriately positioned as adjuncts or supplements to standard care rather than as standalone interventions, particularly for targeting binge eating and emotional eating, as well as depressive and anxiety symptoms [115, 133, 139, 140, 142]. VR-based interventions have shown preliminary potential for promoting weight-loss maintenance, reducing anxiety, enhancing assertiveness, and decreasing body image avoidance; however, most effects were observed within-group, and the evidence base remains limited [116, 137]. Still, findings from this study suggest that the effectiveness of DMHIs may depend not only on the delivery modality but also on key intervention characteristics, such as the therapeutic approach adopted and the level of professional guidance involved. In particular, the effectiveness of digital interventions appears to be greater when programs include therapist- or coach-guided support compared with fully self-guided formats. Videoconferencing platforms, in particular, were associated with minimal attrition, suggesting higher acceptability in synchronous delivery formats. Web- and app-based interventions showed moderate to high dropout rates, yet attrition tended to be lower in programs that incorporated guidance from a therapist or health coach, emphasizing the importance of strategies to enhance adherence in digital health programs. Nevertheless, several self-guided interventions still produced meaningful improvements, indicating that therapist support may not always be essential. Taken together, these findings confirm and extend the growing body of evidence supporting the use of DMHIs for weight management [10, 16, 17]. Despite generally positive outcomes, the acceptability of these interventions remains underexplored, as most studies included in this review did not formally assess acceptability; however, exceptions such as Levin et al. (2021) [126], Yu et al. (2021) [145], and Zamorano et al. (2021) [146] reported favorable participant feedback. Similarly, Brindal et al. (2013) [114] found that their app was well-received and recommended by users. These results highlight the importance of designing DMHIs with structured and monitored intervention components to enhance engagement and facilitate real-world implementation in adults with obesity. A notable gap in the current literature concerns the economic evaluation of DMHIs. Only two studies [127, 138] conducted formal cost-effectiveness analyses, and both suggested that the interventions were unlikely to meet conventional cost-effectiveness thresholds; therefore, cost-effectiveness should be considered an unresolved evidence gap rather than an established advantage of DMHIs. This scarcity of economic evidence represents a significant barrier to the integration of DMHIs into routine healthcare practice and underscores the need for future research to systematically evaluate both the clinical and economic value of these interventions.

## Strengths and Limitations

To our knowledge, this is the first systematic review to evaluate a wide range of DMHIs and therapeutic approaches across multiple outcomes in RCTs involving adults with obesity, allowing for a comprehensive assessment of digital interventions in this population. The exclusion of studies with a high risk of bias further strengthens confidence in the overall pattern of findings. However, several limitations should be considered when interpreting the results. First, the number of studies available for each intervention modality varied considerably, limiting the robustness and generalizability of modality-specific observations. Second, substantial heterogeneity was identified across studies in terms of participant characteristics, intervention content, delivery formats, outcome measures, treatment duration, and follow-up periods. This heterogeneity precluded direct comparisons across intervention types and suggests that findings should be interpreted as indicative patterns rather than evidence of comparative effectiveness. In addition, reporting of implementation-related outcomes was often incomplete. Only a minority of studies provided detailed information on intervention adherence, user engagement, acceptability, or implementation costs, limiting conclusions regarding real-world feasibility and scalability. Potential adverse effects were also infrequently assessed, including possible impacts on disordered eating symptoms, psychological distress, weight stigma, or maladaptive engagement with digital technologies. Similarly, evidence regarding health equity remains limited, as few studies examined whether intervention effects differed according to factors such as digital literacy, socioeconomic status, ethnicity, geographic location, or access to technology. Finally, economic evidence was scarce, with only two studies reporting formal cost-effectiveness evaluations.

## Implications for Future Research

Looking ahead, future trials should adopt rigorous study designs, including larger sample sizes, active comparator groups, and long-term follow-ups, while incorporating comprehensive health economic evaluations to support the sustainable and scalable implementation of DMHIs. Future studies should also prospectively monitor adverse events and unintended effects, including disordered eating symptoms, psychological distress, excessive or compulsive digital engagement, and experiences of weight stigma. Beyond establishing efficacy, studies should systematically assess acceptability and engagement, as well as their determinants, since these factors are critical for real-world uptake and sustained use. Comparative research examining guided versus self-guided DMHIs is needed to clarify the added value of therapist or coach support. Additionally, multimodal interventions that integrate multiple digital tools within a stepped-care framework should be explored, as these may enhance personalization, engagement, and clinical outcomes. Finally, evaluations across diverse levels of digital literacy and socioeconomic contexts are essential to ensure equitable access and benefit. Collectively, these efforts will help advance effective, sustainable, and integrated digital intervention strategies for adults with obesity.

## Guidance for Interventions

**Fig. 2:**
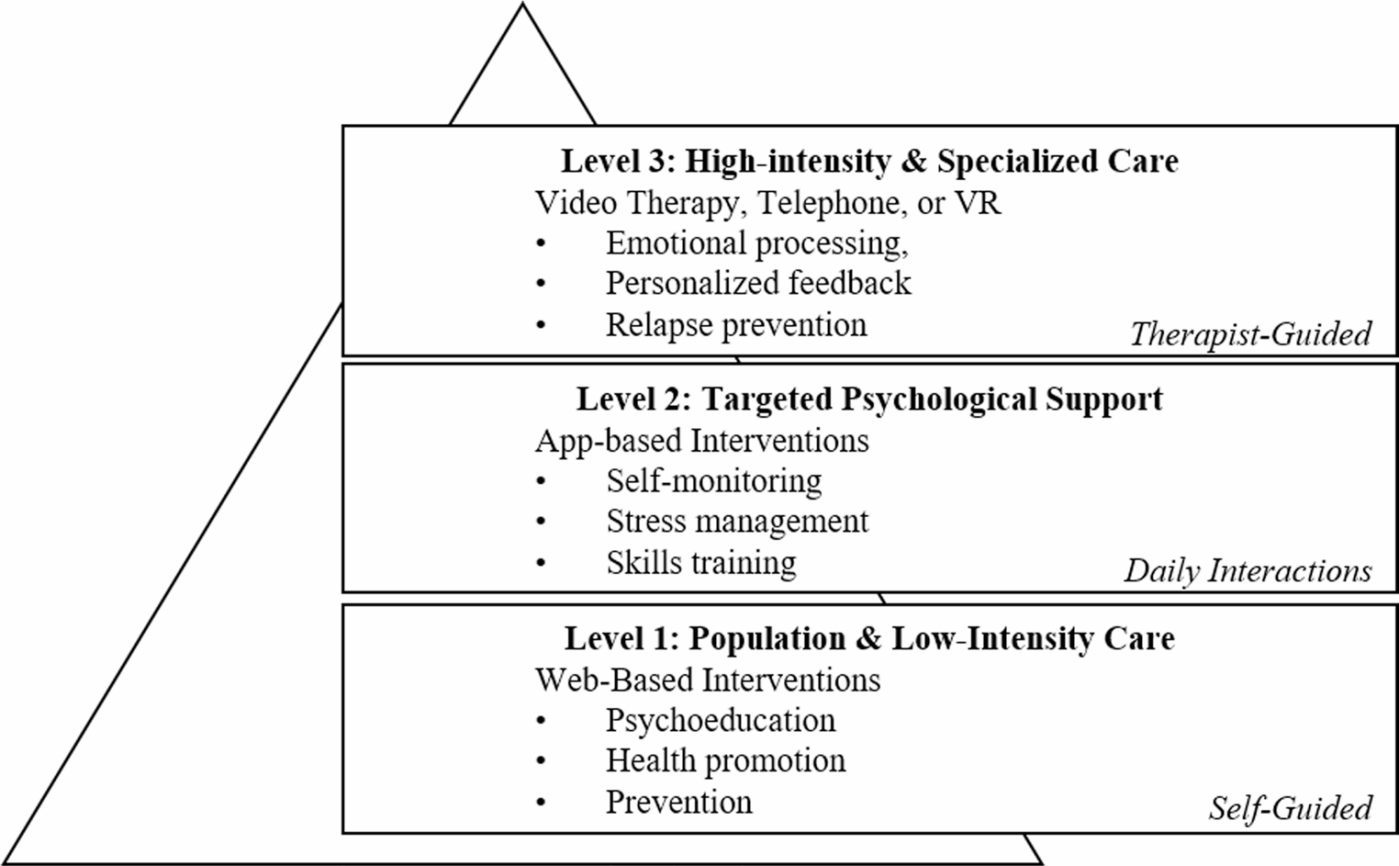
An integrated stepped-care framework for digital obesity treatment

Building on the evidence synthesized in this review and acknowledging that the proposed stepped-care framework is an evidence-informed conceptual model rather than a directly tested comparative model, several recommendations can be proposed to guide the design and implementation of future DMHIs for adults with obesity. Web-based platforms appear particularly efficient for large-scale behavioral activation, health promotion, and dissemination of preventive content at the population level. These interventions consistently demonstrate beneficial effects on weight-related and behavioral outcomes, though their impact on psychological outcomes is more variable. App-based interventions may be especially suited to support psychological flexibility, emotion regulation, and stress management. Their design enables daily, self-paced exercises and ecological momentary support that can be seamlessly embedded into individuals’ everyday contexts, promoting sustained engagement and skill practice. For individuals with more complex psychological needs, including emotional difficulties or body image disturbances, telephone-, videoconferencing-, and VR–based interventions may offer particular advantages. Synchronous modalities that involve real-time interaction with a therapist or coach enable individualized feedback, reinforcement of therapeutic skills, and direct emotional processing, which may enhance both adherence and acceptability. VR-based interventions, by providing immersive and experiential engagement, show promise for addressing body image disturbances and appearance-related distress through controlled exposure, embodiment, and experiential learning techniques. These modality-specific considerations support the adoption of stepped-care and blended care models, in which intervention intensity and delivery format are dynamically tailored to patients’ risk profiles, needs, and response to treatment. At Level 1, interventions predominantly deliver psychoeducation, are self-guided, web-based, and scalable, to promote behavioral activation, health promotion, and preventive strategies suitable for broad dissemination. Level 2 interventions are for individuals at moderate risk or with emerging psychological difficulties. App-based programs at this level can support self-monitoring, skills practice, emotion regulation, stress management, and psychological flexibility, with daily interactions, ecological momentary support, and optional guidance to enhance engagement while maintaining accessibility. At Level 3, interventions are intended for high-complexity cases, non-responders, or relapse prevention. Delivered via videoconferencing, telephone, or VR, these interventions focus on emotional processing, personalized feedback, body image exposure, and maintenance of treatment gains. They are therapist-guided, highly engaging, and individualized, ensuring intensive and tailored care. Across all levels, multimodal integration plays a central role. Combining web-based modules, app-based monitoring, and synchronous therapist support allows interventions to be personalized and adaptive, responding to individuals’ evolving needs, risk profiles, and treatment responses (see Fig. 2).

Overall, adopting a stepped and multimodal delivery model offers considerable potential to optimize outcomes across multiple domains, including weight-related measures, eating behaviors, psychological well-being, engagement, adherence, as well as scalability and equity. Interventions delivered within this framework are likely to enhance both effectiveness and accessibility, promoting a more individualized and responsive approach to obesity management. Future DMHIs should move beyond single-modality approaches and instead integrate multiple digital components to harness their complementary strengths. Hybrid programs that combine web-based psychoeducation, app-based self-monitoring, skills practice, and synchronous therapist contact may improve engagement, facilitate the transfer of skills into daily life, and enhance clinical outcomes, while preserving scalability. While self-guided interventions can produce meaningful benefits, professional guidance should be prioritized for individuals with higher clinical complexity, co-occurring psychological distress, or challenges with adherence. From a policy perspective, these findings highlight the need to move away from default reliance on fully unguided programs and instead allow for flexible integration of therapist or coach support within digital services. Engagement, adherence, and acceptability should be treated as core design targets rather than secondary outcomes. Structured schedules, regular monitoring, and feedback mechanisms appear essential for sustaining participation over time, and systematic assessment of user experience should be routinely incorporated into future research. Finally, policy and implementation decisions must explicitly account for differences in digital literacy and socioeconomic status, ensuring that digital interventions help reduce health inequalities among adults with obesity. Large-scale implementation efforts should also be supported by robust health economic evaluations to assess cost-effectiveness and long-term sustainability.

## Conclusion

DMHIs represent a promising and scalable avenue for addressing the complex interplay of clinical, behavioral, and psychological factors underlying obesity. Their effectiveness appears to vary according to the specific digital format and the level of therapeutic support provided. However, conclusions about the comparative effectiveness of modalities remain tentative because the evidence is heterogeneous and was synthesized narratively. Continued innovation, rigorous evaluation, and integration within stepped-care frameworks are essential to realize their full potential in promoting both mental and physical health in individuals with obesity.

## Key References

- The Lancet: More than half of adults and a third of children and adolescents predicted to have overweight or obesity by 2050 | Institute for Health Metrics and Evaluation. 2025. Available from: https://www.healthdata.org/news-events/newsroom/news-releases/lancet-more-half-adults-and-third-children-and-adolescents [accessed Oct 25, 2025] (of outstanding importance).
  - ○ Offers a current and comprehensive framework for understanding obesity, crucial for designing digital intervention studies and defining the target population.
- Heriseanu AI, Karin E, Walker J, Scott AJ, Bisby MA, Gandy M, Dudeney J, Fisher A, Titov N, Dear BF. The impact of obesity and overweight on response to internet‑delivered cognitive behavioural therapy for adults with chronic health conditions. Int J Obes 2023 June;47(6):487–495. doi:10.1038/s41366-023-01285-6 (of outstanding importance).
  - ○ This study provides robust empirical evidence on how overweight and obesity influence treatment response to internet-delivered cognitive behavioral therapy in individuals with chronic conditions.
- Irvin L, Madden LA, Marshall P, Vince RV. Digital Health Solutions for Weight Loss and Obesity: A Narrative Review. Nutrients 2023 Apr 12;15(8):1858. doi: 10.3390/nu15081858 (of importance).
  - ○ Comprehensive review of DHI for obesity, summarizing behavioral and psychological outcomes.
- Monreal-Bartolomé A, Castro A, Pérez-Ara MÁ, et al. Efficacy of a Blended Low-Intensity Internet-Delivered Psychological Program in Patients With Multimorbidity in Primary Care: Randomized Controlled Trial. J Med Internet Res 2025 Feb 10;27:e56203. doi: 10.2196/56203 (of importance).
  - ○High-quality RCT demonstrating effectiveness of low-intensity, blended digital interventions in populations with complex health conditions, directly translatable to obesity management.

## Supplementary Information

Below is the link to the electronic supplementary material.

Supplementary Material 1 (PDF 45.8 KB)

Supplementary Material 2 (XLSX 20.0 KB)

Supplementary Material 3 (XLSX 20.0 KB)

Supplementary Material 4 (XLSX 24.0 KB)

Supplementary Material 5 (XLSX 32.0 KB)

## Data Availability

No datasets were generated or analysed during the current study.
